# Testing the feasibility, acceptability, and exploring trends on efficacy of the problem management plus for moms: Protocol of a pilot randomized control trial

**DOI:** 10.1371/journal.pone.0287269

**Published:** 2024-01-05

**Authors:** Irene Falgas-Bague, Maria Melero-Dominguez, Daniela de Vernisy-Romero, Thandiwe Tembo, Mpela Chembe, Theresa Lubozha, Ravi Paul, Doug Parkerson, Peter C. Rockers, Dorothy Sikazwe, Günther Fink

**Affiliations:** 1 Department of Epidemiology and Public Health. Swiss Tropical and Public Health Institute, Basel, Switzerland; 2 Faculty of Economics, University of Basel, Basel, Switzerland; 3 Disparities Research Unit, Mongan Institute, Massachusetts General Hospital, Boston, Massachusetts, United States of America; 4 Department of Medicine. Harvard Medical School, Boston, Massachusetts, United States of America; 5 Department of Global Health. Boston University School of Public Health, Boston, Massachusetts, United States of America; 6 Chainama Hills Hospital, Lusaka, Zambia; 7 Innovations for Poverty Action, New York, New York, New York, United States of America; 8 Department of Psychiatry, University of Zambia, Lusaka, Zambia; 9 Zambia Ministry of Health, Lusaka, Zambia; University of Nicosia, NEPAL

## Abstract

Mental health disorders are one of the most common causes that limit the ability of mothers to care for themselves and for their children. Recent data suggest high rates of distress among women in charge of young children in Zambia. Nevertheless, Zambia’s public healthcare offers very limited treatment for common mental health distress. To address this treatment gap, this study aims to test the feasibility, acceptability, and potential efficacy of a context-adapted psychosocial intervention. A total of 265 mothers with mental health needs (defined as SRQ-20 scores above 7) were randomly assigned with equal probability to the intervention or control group. The intervention group will receive a locally adapted version of the Problem-Management Plus and “Thinking Healthy” interventions developed by the World Health Organization (WHO), combined with specific parts of the Strong Minds-Strong Communities intervention. Trained and closely supervised wellbeing-community health workers will provide the psychosocial intervention. Mental health distress and attendance to the intervention will be assessed at enrollment and 6 months after the intervention. We will estimate the impact of the intervention on mental health distress using an intention-to-treat approach. We previously found that there is a large necessity for interventions that aim to address mother anxiety/depression problems. In this study, we tested the feasibility and efficacy of an innovative intervention, demonstrating that implementing these mental health treatments in low-income settings, such as Zambia, is viable with an adequate support system. If successful, larger studies will be needed to test the effectiveness of the intervention with increased precision.

**Trial registration:** This study is registered at clinicaltrials.gov as NCT05627206.

## Introduction

Mental health disorders are a major global health issue and a significant cause of disability [1]. Depression and anxiety, also known as common mental health disorders (CMHD), are the most prevalent mental health disorders, with approximately 300 million cases worldwide in 2019, affecting women in greater proportion than men [2, 3]. CMHD result from a complex interaction of social, psychological, and biological factors, and their development is related to chronic stress exposure as well as to acute trauma experiences. Ever since the beginning of the COVID-19 pandemic there has been an increasing concern about the negative effects of the pandemic on individuals’ mental health, with the greatest effect seen among women living in under-sourced contexts, such as low- and middle-income countries (LMICs) [4]. Among women living in LMICs, mothers in charge of children under five years old are considered a key group to study and intervene in [5], as CMHD may limit not only their ability to care for themselves, but may also subsequently impact their children’s development, contributing to the South-North poverty gap [6–8].

The COVID-19 pandemic has increased the risk of developing CMHD among mothers living in sub-Saharan Africa [5]. In Zambia, women primarily responsible for caring for young children were already suffering from high rates of distress, and the COVID-19 pandemic and its consequences in society (e.g. loss of jobs, closure of markets, reduction of healthcare visits) have exposed them to even higher levels of stress. Recent findings suggest that 26.1% of women in charge of young children suffer from mental health distress and that younger and less educated mothers were the most vulnerable during the COVID-19 pandemic, with higher mental health deterioration [5]. Despite the high rates of mental health needs, CMHD usually remain unnoticed and mainly untreated in this setting [9].

Undetected and untreated mental health problems adversely affect women and their children in many ways, for example, by reducing well-being and quality of life, increasing food insecurity, and deteriorating mother-child relationships. Altogether, mothers’ mental health distress can result in inhibited child development and an increased risk of anxiety, reduced physical growth, cognitive impairment, and higher risks of non-adherence to HIV treatment for both mother and child [10].

Zambia faces several challenges in addressing CMHD within its primary care system. Shortage of mental health professionals, unmet training needs, and lack of evidence-based psychosocial interventions adapted to the local context are currently the most important obstacles [9, 11, 12]. Effective treatments for mental health disorders are widely available in high-income countries, but rarely applied in LMIC settings. In fact, the public healthcare system in Zambia, similar to other neighboring LMICs, offers very limited mental health services at the primary healthcare and community levels. The existing mental health workforce is concentrated in a few psychiatric institutions and high-level hospitals, which are permanently full and focus on attending people with severe mental illnesses [13].

Evidence suggests that culturally adapted psychosocial programs, delivered by trained paraprofessionals or community health workers, can effectively reduce CMHD burden [14, 15]. Community-based psychosocial programs increase mental healthcare access while providing treatment of CMHD within the community, reducing the work burden of the specialized mental health workforce while increasing workforce capacity [16, 17]. Specifically, the Problem Management Plus, a World Health Organization transdiagnostic intervention, has been shown to improve mental health outcomes and psychosocial functioning while also being cost-effective [18]. The intervention has proven to be feasible and acceptable not only when implemented in high-income settings [19, 20], but also in various LMICs and among diverse populations [21, 22]. However, to our knowledge there are no culturally relevant treatments adapted to women in charge of young children living in low-income settings, which may reduce the engagement of these interventions as well as their impact [23]. Targeted programs using evidence-based practices should consider the main obstacles that women face in engaging in these interventions (i.e. domestic duties and lack of autonomy) and include topics that are specifically of women’s interest (i.e. self-care routines, parenting activities and/or empowerment strategies).

As such, we conducted a randomized clinical trial testing the feasibility, acceptability and potential efficacy of a culturally adapted “Problem-Management-Plus-For Moms” program with a sample of mothers residing in the Lusaka district of Zambia.

## Methods

### Aims of the study

The overall objective of this project is to assess the feasibility, acceptability, and explore the efficacy of the adapted Problem-Management-Plus-For-Moms (PM+FM) on mothers’ wellbeing in a representative sample of the urban Zambian community. To achieve this goal we will:

Specific Aim 1: Assess the mothers’ acceptability of the PM+FM intervention (measured as >50% of sessions attended, satisfaction measure and qualitative feedback).

Specific Aim 2: Assess the feasibility of the PM+FM intervention within an urban Zambian context (measured as ≥ 1 session attended and offered and participants’ and providers’ qualitative feedback).

Specific Aim 3: Explore any trends in efficacy of the PM+FM intervention on mothers’ mental health status (measured as percentage of women improving SRQ scores).

Additionally, we will assess any potential change in the mother-child relationship using the World Bank Toolkit for early child development.

### Study design

**Fig 1:**
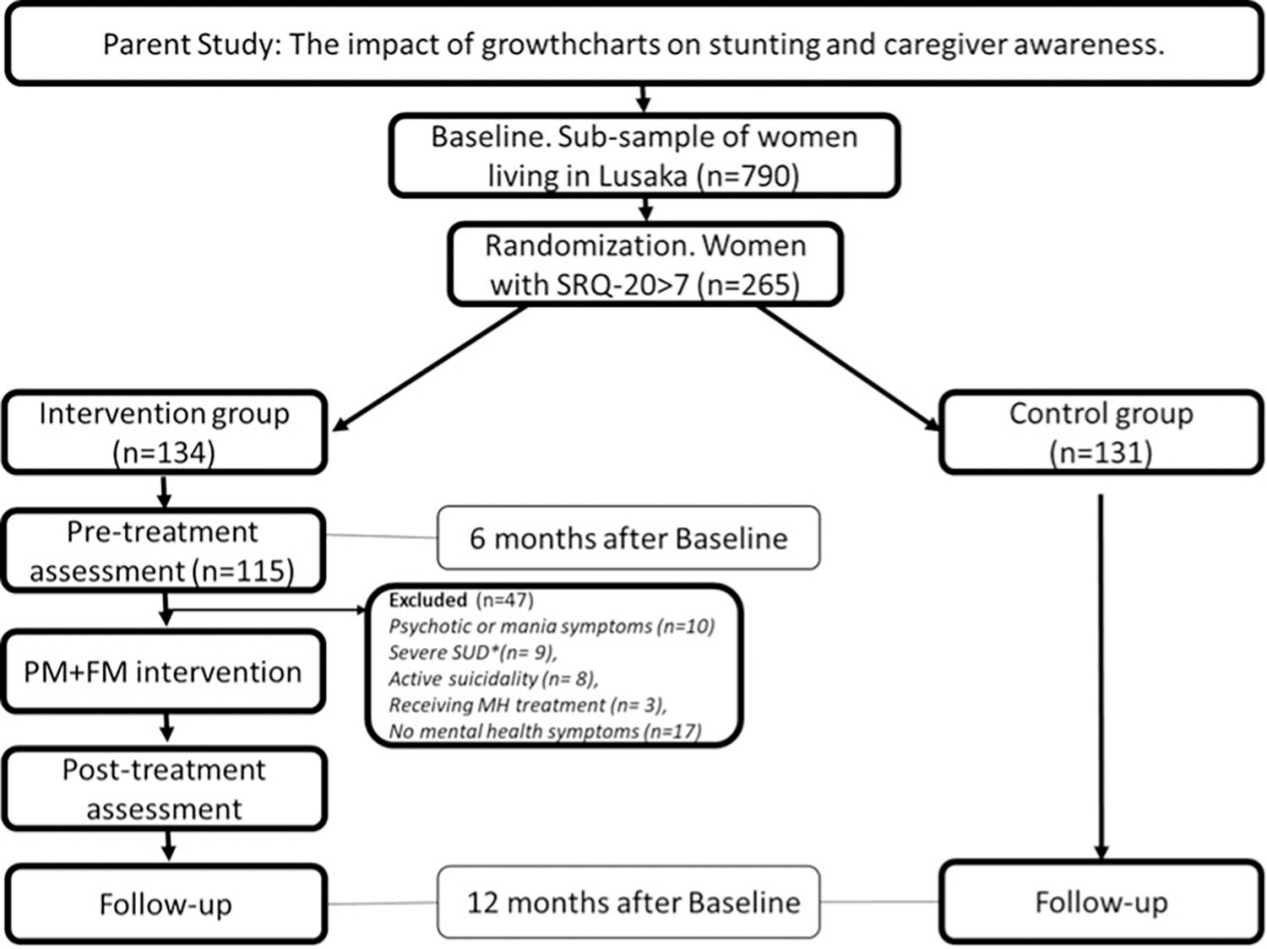
Flowchart of study design. Flowchart showing participant recruitment and enrollment, as well as details of study design.

This is a randomized clinical trial nested within a parent trial testing the impact of a child-focused intervention (based on providing information about nutrition and/or nutritional supplements to babies) on children’s growth, the Zamcharts trial (NCT0512042). To assess eligibility for the PM+FM study, we used data from the 790 women enrolled in the parent trial and living in Lusaka. Fig 1 shows the participant flowchart and study design details.

### Study setting and participants

#### Study setting

This study was conducted in the Lusaka district of Zambia. The Lusaka district is an urban district, which includes the capital, the most populated city in the country, and its surrounding neighborhoods. The Lusaka district is characterized for its ethnic diversity, hosting people from different areas of the country. Most of the health resources and services provided involve access to tertiary hospitals and primary care facilities.

#### Eligibility criteria

The eligibility criteria for this study were 1) adults (18 years+) enrolled in the Lusaka subsample of the larger study (ZamCharts parent study), 2) having a high SRQ at baseline (SRQ>7) and 3) not receiving mental health care during the re-assessment for this study.

All selected women from the intervention group were contacted and reassessed with the Self-Reporting Questionnaire (SRQ-20) and the Hopkins Symptoms Checklist 25.

#### Exclusion criteria

Females without capacity to consent, with active suicidal ideation (determined by Paykel scale with scores 4 and/or 5) [24], severe substance use (AC-OK: ADULT Screen for Co-Occurring Disorders, Substance Abuse; ACOK-SUD >4) [25], or mania or psychotic symptoms were excluded and referred to a partnering psychiatrist for further evaluation and treatment [26]. Additionally, women planning to move in the next 6 months were excluded from the study.

#### Recruitment, assessment, and engagement procedures

Trained interviewers blinded by the treatment condition assessed women’s final eligibility (see Fig 1 and eligibility criteria for exclusion criteria), minimizing the risk of reporting or social desirability biases. Interviewers explained the nature of the study to the participants and answered all questions prior to obtaining written informed consent.

We started with the identification of cases using the baseline data from the parent trial, which enrolled all mothers with a 6–11 month old child in 90 randomly selected enumeration areas of Lusaka (1^st^ assessment point conducted in June 2022). Then, the research assistants contacted women by phone and scheduled in-person assessments to ensure eligibility to participate in the intervention. Enrollment was completed by December of 2022. Assessment for eligibility and consent took an average of 1h and 10 minutes. A post-treatment assessment was conducted among participating mothers approximately five months after the initial eligibility survey (May 2023), immediately after completing the intervention. A final assessment with all 265 study participants will be conducted 12 months after baseline (June-September 2023). We aim to complete data collection and conduct all analyses by December 2023.

We focus on engaging participants in each of the research components of the trial (assessments and sessions) through a systematic engagement protocol including regular calls to participants and home visits after two weeks of non-responsiveness. All participants are given a small incentive (i.e. a piece of soap) at the end of each interview assessment. No incentives will be provided to attend to the intervention sessions.

#### Randomization of participants

All eligible subjects (n = 265) were randomized with a probability of 0.5 to either the treatment or the control group. Randomization is based on a random draw generated by the senior author (GF) using the Stata SE 16.0 statistical software package.

### Treatment of participants

#### Experimental intervention

Participants in the intervention group (n = 134) were offered the PM+FM intervention program delivered by WCHWs. The intervention has been developed specifically to target mothers of young children living in sub-Saharan Africa. It combines elements from the PM+ intervention and “Thinking Healthy” intervention developed by WHO [18, 27] with some selected components of the Strong Minds-Strong Communities psychosocial intervention [28].

To develop the PM+FM intervention, we followed the Castro-Barrera framework for cultural adaptation of evidence-based interventions [29]. First, we gathered evidence from pre-existing published information on delivering psychosocial interventions in low-income settings, cultural expressions of depression and anxiety symptoms among women in sub-Saharan Africa, and main sociocultural stressors among women. We complemented this literature review with two focus groups and three in-depth interviews with women with small children in Zambia. Second, the core components from the PM+ were combined with elements from the “Thinking Healthy” manual and specific sections from the Strong Minds psychosocial intervention (e.g. psychoeducation about healthy habits). We adapted the language use and added notes for the WCHWs with tips on how to implement each strategy and specific local language wording they could use, so that the WCHWs (with basic training in mental health) could effectively deliver the intervention. Third, we incorporated the feedback from the research team, local mental health experts, and from trained WCHWs. For example, we included a case example to increase comprehension of the different strategies and relevance to the targeted group (i.e. a case vignette representative of a Sub-Saharan woman that is presented with a new “problem” each session and a collaborative practice using the session’s new strategy). Finally, we pilot tested the intervention manual with a small group of individuals with mild symptoms and incorporated their final feedback in the last version of the manual. The final intervention, PM+FM, is culturally adapted to women’s local context of social and cultural values and norms. It is a cognitive-behavioral therapy-based transdiagnostic psychosocial intervention aiming to improve mothers’ well-being, including depression and anxiety symptoms and mother-child relationships. It follows a collaborative and patient-centered approach where an individual’s needs are central, and contents of the intervention are adapted to each person.

In comparison with the original PM+ that consists on five individual sessions lasting one hour and a half, the PM+FM intervention is provided mostly individually (sessions 1 and 10 can be delivered in groups of a maximum of 8 women, but sessions 2 to 9 are delivered individually). It is organized in ten 1-hour sessions, and provided on a weekly basis by a specifically trained and supervised WCHW in women’s homes and/or by phone. Group sessions are provided in community healthcare centers close to their residence. The program lasts a maximum of 4 months and combines psychoeducation, problem-solving and cognitive restructuring techniques, motivational interviewing approaches, effective communication, and mindfulness practices. Table 1 shows the main components of the PM+FM intervention.

**Table 1:** Intervention summary of contents.

PM+FM Intervention Summary of Contents	
Session 1	Symptoms assessment. Introduction of the program and confidentiality. Cultural Formulation interview to understand women’s needs. Psychoeducation about mental health needs (understanding adversity) and maternal-child positive relationship. Relaxing breathing.
Session 2	Barriers to change, managing problems and stress management strategies. Introduction to mindfulness.
Session 3	Symptoms assessment. Managing Problems (Problem-solving technique), “get going, keep doing” strategies (Behavioral activation). Managing Stress strategy and mindfulness practice.
Session 4	Managing Problems: understanding unhelpful thoughts. Positive upbringing strategies. Stress management and mindfulness practice.
Session 5	Symptoms assessment. Importance of support for positive upbringing and stress management. Strengthening social support. Mindfulness practice.
Session 6	Effective communication skills, Mindfulness practice.
Session 7	Symptoms assessment. Improving healthy habits for me and my child (nutrition, sleep, and substance use). Mindfulness practice.
Session 8	Positive thinking and self-compassion practice. Mindfulness practice.
Session 9	Review of the techniques. Staying Well and Looking to the future (Self-care plan). Mindfulness practice.
Session 10	Review of a self-care plan. Social resources and referral. Mindfulness practice. Closure and Ending the program.

Women with severe mental health symptoms or with severe substance use symptoms were referred to our collaborating team of psychiatrists and psychologists from the University Teaching Hospital and Chainama Hospital in Lusaka. Medication prescription and monitoring was tracked by the research team. Pharmacological treatment (indicated by the psychiatrist) was subsidized by the project, in case participants cannot afford it.

#### Training and supervision

We identified ten people with at least a high school diploma, fluent in English and the main local language (Nyanja), with previous experience in working within the community and interested in mental health topics. After an introductory meeting, the research team selected five of the identified people to continue with the training and to participate as WCHWs. Selection was based on their interest in the job, performance in a basic skills assessment, and availability. The training program entailed 4 full days of instruction followed by 4 weeks of role-plays with a 2-hour weekly group supervision. The PI of the trial, a local psychiatrist member of the research team and a local psychologist, performed the instructional training mostly in English. Instruction started by addressing program objectives, ethics, emergency protocol procedures, psychoeducation about common mental health problems (Day 1) and basic components of psychotherapy (i.e., motivational interviewing, cultural formulation, mindfulness practice and problem-solving techniques) (Day 2). The third and fourth days of instructional training focused on the specific sessions’ content, special clinical situations (i.e., substance use involvement, domestic violence, and suicidal thoughts), and training related to their integration in the treatment site, including working at participant homes, communication, and referral procedures. It ended with a review of the emergency protocol procedures. The training expanded on practical examples related to common issues within the urban-Zambian cultural background of the participants. These specific parts of the training were conducted in Nyanja. Instructional trainings were video recorded for further use in subsequent trainings. Since the manual has been developed in English, to support and uniform the intervention provision in the local language, the research team along with the WCHWs co-created a glossary including the most important concepts (e.g. anxiety, stress, unhelpful thoughts, worries) used during the sessions. After completing a first roleplay (including 10 sessions), in which one sessions were supervised by the PI, the WCHWs completed a second set of roleplays in the local language and received weekly supervision by the local clinicians. Once the clinical supervisors approved their performance, WCHWs were assigned with 10 to 15 cases each. All role-plays and the first two cases’ sessions were audiotaped and heard by the supervisors. The supervision protocol includes weekly individual and group supervision throughout the training process (individual supervision) and trial (group supervision) to support the WCHWs and to provide feedback on the session tapes, share the fidelity forms and to resolve issues related to the delivery (e.g., severe cases and drifting). We plan to collect data on training needs and on the basic knowledge of mental health among the WCHWs at the moment of hiring and every 6 months, to evaluate mental health care delivery capacity. The supervisors are local psychologists and a psychiatrist specially trained by the PI of this project.

The proposed intervention does not pose any known risk to the women participating. Similarly, if women are not interested in being part of the intervention, they can simply inform the study staff that they are no longer interested.

#### Comparator

The comparator (control group) received no intervention. Participants included in our study are ensured access to a women’s healthcare network for assistance.

## Measures and outcomes

### Primary endpoints

Feasibility will be assessed by the percentage of participants completing 1 or more sessions of the intervention and through a focus group with the intervention providers.

Acceptability will be evaluated using the percentage of participants completing the program (≥ 50% sessions completed) together with qualitative feedback from participants and providers through a semi-structured interview at the end of the intervention and participants’ feedback from a semi-structured interview at the end of the intervention. To explore the effectiveness of the intervention, our primary endpoint will be SRQ-20 ≤ 7, 12 months after baseline (approximately 3 months after completing the intervention), comparing control and intervention women.

### Secondary endpoints

The secondary endpoints are the mental health distress post-treatment (measured by Hopkins Symptom Checklist-25 (HSCL-25) <1.06) [30–32], psychological outcome profiles to measure women’s wellbeing (PSYCHLOPS) [33], mother-child interactions measured by a selected measure from the World Bank Toolkit to address mother-child interactions and child early development [34], and functionality through World Health Organization disability assessment schedule 2.0 12-Item Survey (WHO-DAS 2.0) [35]. Table 2 displays measurement details, including validation data. Additionally, S1 Fig from the supporting information displays temporal data for each measurement.

**Table 2:** Outcomes.

Outcomes	Measures Exploratory Efficacy Outcome
**Primary Outcome Measures for PM+FM pilot trial**	
**Feasibility**	Percentage of 1 or more sessions completed. Completion rates; ease/burden. Qualitative feedback from participants and providers.
**Acceptability**	1) Participation rates; 2) Satisfaction scale; 3) Relevance of PM+FM. Qualitative feedback from participants and providers.
**SRQ-20**	20 items measuring risk of common mental health problem. Used in the intent-to-treat analysis to explore effectiveness of the intervention using control group (12-months follow up data from parent study). Cut off of >7 will determine presence of mental health distress based on previous validation studies based in similar context [36]. Internal consistency from a similar sample in the same context, α = 0.90 [5].
**Secondary Outcomes (pre- post- analysis)**	
**HSCL-25**	10 questions about anxiety, 15 questions about depression; Internal consistency from the adapted test using our sample at baseline (α = 0.92). Cut-off used at 1.06 based on validation in similar samples [32].
**WHO-DAS 2.0**	12 questions related to disability and functionality. Internal consistency from the adapted test using our sample at baseline, α = 0.69.
**World Bank’s Toolkit and Inventory**	Selected measures from the World Bank Toolkit to address mother-child interactions and child early development.

SRQ-20: Self-Report Questionnarie; HSCL-25: Hopkins Symptom Checklist-25; WHO-DAS 2.0: WHO Disability Assessment Schedule 2.0.

### Adaptation of measures to the local context

We prioritized the use of questionnaires that were previously used in a similar context [36–38]. Questionnaires were translated into the main local language (Nyanja) using a stepped process following International Test Commission guidelines [39]:

Formal Translation: first, the questions were translated into Nyanja by a professional translation firm with experience in health-related translations and using a translation and back-translation process. The translators were native speakers of Nyanja and fluent in English. They were instructed to use simple and clear language that would be easily understood by the target population.

Native speakers review: a native Nyanja-speaking research member and the group of WCHWs who were trained to provide the intervention reviewed the translated and back-translated questions. The panel evaluated the semantic, conceptual, cultural, and linguistic equivalence of the questions and suggested modifications or replacements for items that were inconsistent, translation errors, or that were not culturally appropriate or relevant to the target context.

Pre-test: The revised questions were pre-tested with a sample of 10 participants who were not eligible for the intervention. They were asked the same questions to gauge the ease of the comprehension, clarity, relevance, and acceptability. Based on the feedback from the pre-test, the questions were finalised and approved by the research team. Any translation errors or inconsistencies were corrected.

Pilot testing: The final version of the questions was administered to a pilot sample of 50 participants. Their responses were collected and analyzed by the PIs to assess the validity and reliability of the questions. The psychometric properties of the questions were evaluated. The final version of the questions was formatted and prepared for administration.

### Collection of qualitative data

During the post-treatment assessment, trained research assistants conducted open-ended questions to participants randomized to the intervention arm to gather their direct opinion regarding the feasibility, acceptability, and barriers for implementation of the PM+FM in a larger trail. Two questions assessed feasibility by asking about difficulties in completing the first session of the intervention and what was found most helpful of session 1. For acceptability, we focused on questions related to understanding the engagement to the program. We also assessed overall feedback to uncover obstacles on intervention implementation, seek for potential mediators of the intervention (e.g. relationship with the WCHW), and develop explanatory models of the effectiveness results.

To complement participant’s data, members of the research team held two focus groups with the trained WCHWs that participated in the intervention provision (n = 5) to gather further information about feasibility, acceptability and the implementation of the PM+FM, including the training of the WCHWs, the materials, and the session lengths. Focus groups followed a semi-structured interview guide with questions such as *“if you could change any aspects of the intervention*, *what would they be*?*”*.

### Data management system

All data will be collected and stored electronically using the Survey Collect (SurveyCTO) software. Survey CTO is tool intended to facilitate mobile data collection services. It consists of an Android app that replaces paper forms used in survey-based data gathering. It supports a wide range of questions and answer types and can be utilized without network connectivity. All data collection will occur offline on password protected Android devices. Data connectivity will only be used to send data collected in the field. Data storage will be on a local secured computer in Zambia and on ServeyCTO’s secure servers.

#### Data security, access, archiving and back up

Project data is only accessible to authorized personnel who require data to fulfill their duties within the scope of the research project. On all of the documents, participants will only be identified by a unique participant number. Data and databases will not be shared with the public and will only be available (fully anonymized) for verification purposes of authorities or scientific journals (as a condition to publish results)–upon request only. All study data will be archived for a minimum of ten years after study completion.

### Statistical analysis

First, we began with descriptive analyses of all data by group, examining the balance between treatment and control groups (Table 3).

**Table 3:** Baseline characteristics from the enrolled participants (n = 265).

	Control		Treatment	
	N = 131		N = 134	
	M	SD	M	SD
Caregiver age	27,65	8,35	27,49	6,79
Caregiver years of schooling	3,11	1,08	3,09	1,10
Boys under age 5	0,81	0,75	0,78	0,72
Girls under age 5	0,85	0,78	0,85	0,67
Children under care	2,90	1,30	2,80	1,30
Household members	6,50	2,66	6,38	2,32
Asset quintile	2,68	1,51	2,61	1,49
SRQ score	12,08	3,13	12,07	2,93

*M: Mean; SD: Standard Deviation.

Once all of the intervention data has been collected, we will proceed with a pre-post intervention analysis in the treated sample only, using the Hopkins Symptoms Checklist as a main outcome, to allow for the comparison of mental health symptoms before and after receiving the intervention.

The feasibility, acceptability, and difficulties and successes in carrying out research and intervention activities will be explored through a mixed-methods analysis including dosage analysis (attendance to the intervention’s sessions), semi-structured interviews with the participants, and focus group with intervention’s providers. The burden of completing the assessments and PM+FM on the time and effort of participants, satisfaction with the intervention, and barriers and facilitators to adherence will be explored through semi-structured interviews with all the participants enrolled in the intervention arm (including participants that have dropped out). Qualitative data resulting from the open-ended questions to participants and focus groups with WCHWs will be transcribed, coded and analysed using a thematic approach to inform on feasibility, acceptability, and to explain the potential efficacy of the PM+FM intervention.

To explore effect sizes and trends of efficacy between the intervention group and the non-treated group from the parent trial, we will use the endline data from parent trial. We will use standard linear regression models to assess the impact of the intervention arm on women’s mental health. We will include WCHW and group fixed effects in our empirical model to explore and correct for potential correlation in outcomes. The primary outcome variable will be SRQ-20, used as a dichotomous variable with the cutoff at seven. Separate models will be estimated with and without baseline covariates. Our primary model will be estimated following an intent-to-treat approach. We will also estimate complementary per-protocol models restricting the intervention group to women completing the core components of the intervention (≥ 6 sessions).

#### Interim analyses

No interim analysis is foreseen.

#### Deviations from the original statistical plan

Any deviations from the original statistical plan will be reported in the Methods section of the trial paper.

#### Handling of data

Missing data will arise when a participant refuses to answer a question or complete part of the examination. Missing data may also arise if a participant drops out of the study. All participants are informed that their participation is completely voluntary and that they may refuse to answer any of the questions asked and can stop the evaluation at any time as well as discontinue participation at any time without any consequences. Missing data on covariates will be imputed using multiple imputations using chained equations. Missing outcome data due to sample attrition will not be imputed; however, we will conduct and report analyses examining the extent to which attrition differed across treatment conditions.

#### Sample size calculation

Based on our research team’s previous study [5], 30% of the women enrolled in the parent trial displayed mild to severe symptoms of depression and/or anxiety (SRQ-20 >7). For this study with 790 women from the parent trial residing in Lusaka, approximately 240 should present mild to severe symptoms. With an expected analytical sample size of 100 women per arm, we are powered to detect an intent-to-treat difference of 20 percentage points in the prevalence of depression at endline with power 0.8, and 23 percentage point difference in depression with power 0.9. We are aware that the current trial is not powered to detect a smaller effect size. Thus, we will only explore the efficacy of the intervention to generate some preliminary efficacy estimates that will serve us to power and elucidate the needed sample size for a larger effectiveness trial.

### Datasets to be analyzed and analysis populations

The main analysis will primarily rely on the data collected in the 12 month survey. Baseline variables will be used to 1) assess the extent to which balance was reached through the randomization and 2) to create a set of control variables used in adjusted models.

### Participant confidentiality

All records will be collected electronically and stored on a secure server. For the analysis, all personal information will be removed, and a unique Participant Number (e.g. 2738) will be assigned to each participant. The Investigators will keep a separate confidential enrollment log that matches identifying codes with the participants’ names and residencies, preferably in the Trial Master File.

### Ethics, audits, and inspections

The study protocol (available at S1 File) follows the SPIRIT guidelines for protocol reporting (S1 Checklist) and has been approved by the ethical committees from Northern-Eastern region of Switzerland (AO_2021–00016) and the University of Zambia (1411–2020). Participants excluded for symptom severity were referred to psychiatric care after clinical evaluation. All participants in the control arm were ensured access to women’s healthcare services for continuity of care. The study documentation and the source data/documents are accessible to the ethics committees and auditors/inspectors at all times. All involved parties will keep participant data strictly confidential.

## Discussion

### Dissemination of results and publication policy

#### Dissemination to the scientific community; include lead in publications

Data analysis will lead to a series of synthesized reports and publications in peer-review scientific journals, for which all principles of data safety and protection are considered, and any results are presented in a fully anonymized manner.

#### Information of community and policymakers

This study will be conducted in close collaboration with the Zambian Ministry of Health (MoH). We will present the results of this trial to the mental health office of the MoH (in their quarterly meetings) and to an advisory board formed by a local team of clinicians and policymakers.

## Supporting information

S1 Checklist SPIRIT 2013 checklist: Recommended items to address in a clinical trial protocol and related documents*. (DOCX)

S1 Fig Spirit. (TIF)

S1 File Clinical study protocol. (DOCX)
