# Supplementary figures and images for: Testing the feasibility, acceptability, and exploring trends on efficacy of the problem management plus for moms: Protocol of a pilot randomized control trial
Source: PLoS One. 2024 Jan 5;19(1):e0287269. doi: 10.1371/journal.pone.0287269 (PMC10769019; doi:10.1371/journal.pone.0287269)

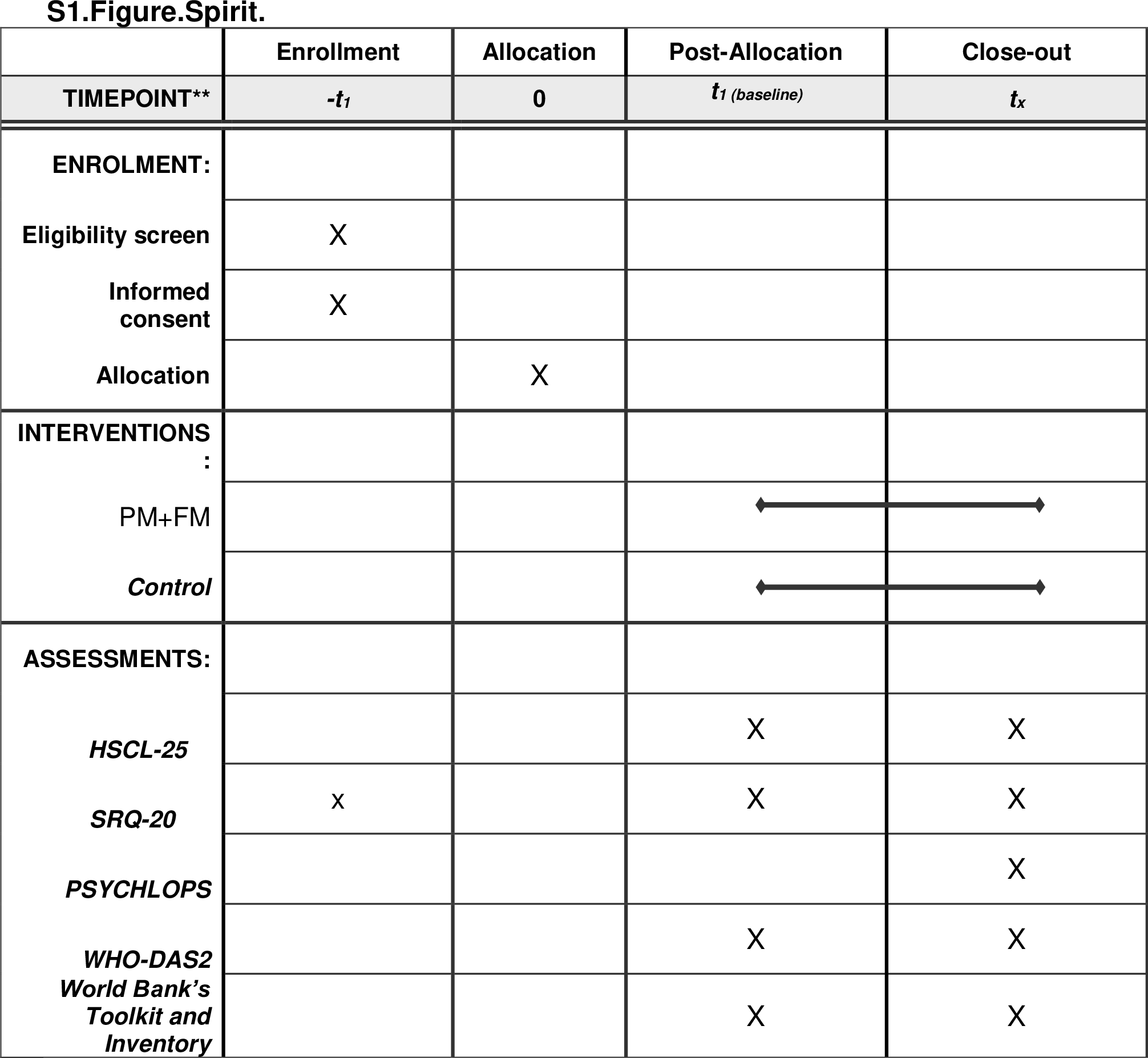

Supplement: S1 Fig — (TIF) [file pone.0287269.s002.tif]
