# Supplementary material for: Testing the feasibility, acceptability, and exploring trends on efficacy of the problem management plus for moms: Protocol of a pilot randomized control trial
Source: PLoS One. 2024 Jan 5;19(1):e0287269. doi: 10.1371/journal.pone.0287269 (PMC10769019; doi:10.1371/journal.pone.0287269)
Supplement: S1 File — (DOCX) [file pone.0287269.s003.docx]

**Clinical Study Protocol**

| **Protocol** | Problem Management + For Moms (PM+FM) | | |
| --- | --- | --- | --- |
| **Version Number** | 1.1 | **Document Date** | 29.10.2022 |
| **Study Registration** | This study will be registered at clinicaltrials.gov. | | |
| **Sponsor Contact** | Günther Fink Socinstrasse 57, 4051 Basel;  061-284-8354;  [guenther.fink@swisstph.ch](mailto:guenther.fink@swisstph.ch) | | |
| **Principal Investigators** | Günther Fink Socinstrasse 57, 4051 Basel;  061-284-8354;  Irene Falgas-Bague  Kreutzstrasse 2, 4123 Allschwil;  41 784055878 | | |
| **Investigational medicinal product** | Not applicable | | |

1. **General Information**
2. **List of Investigators and other persons involved**

| **Title** | **Names** | **Institution** | **Position** | **Function in study** |
| --- | --- | --- | --- | --- |
| Associate Professor/Head of Research Unit | Günther Fink | University of Basel and Swiss TPH | Principal investigator | Dr. Fink will lead and coordinate the overall project, with a particular focus on study design and data analysis. with the co-PIs |
| Dr. | Irene Falgas Bague | University of Basel and Swiss TPH | Co- Principal Investigator | Dr. Falgas Bague will lead and coordinate the overall project with the co-PIs |
| Full Professor | Ravi Paul | University of Zambia | Co-Investigator | Dr. Paul will be the local head clinician and investigator. Will review the study documents and receive the mental health referrals |
| Mr. | Doug Parkerson | Innovations for Poverty Action | Co-investigator | Mr. Parkerson will co-lead all aspects of the project, with a particular focus on field work coordination. He will also handle donor interactions. |

1. **Signatures**

Principal Investigators

- I have read this protocol and agree that it contains all necessary details for carrying out this study. I will conduct the study as outlined herein and will complete the study within the time designated.
- I will ensure that all individuals and parties contributing to this study are qualified and I will implement procedures to ensure integrity of study tasks and data.
- I will provide copies of the protocol and all pertinent information to all individuals responsible to me who assist in the conduct of this study. I will discuss this material with them to ensure they are fully informed regarding the intervention proposed herein and the conduct of the study.
- I will use only the informed consent forms approved by the Sponsor or its representative and will fulfil all responsibilities for submitting pertinent information to the Independent Ethics Committees responsible for this study.
- I agree that the Sponsor or its representatives shall have access to any source documents from which Case Report Form information may have been generated.
- I agree to conduct the study in compliance with the current version of the Declaration of Helsinki, KFPE principles for transboundary research as well as all national legal and regulatory requirements.

**Principal Investigator**

| Signature |  | 1 August 2022 |
| --- | --- | --- |
| Name | Irene Falgas-Bague Gunther Fink | Date of Signature |
| Title | Post-Doctoral scientific collaborator/ Associate Professor/ Head of Research Unit | |
| Institution | University of Basel and Swiss TPH | |
| Address | Kreutzstrasse 2, 4123 Allschwil | |
| Phone | 061-284-8354 | |

1. **Table of contents**

[2. Background information 10](#_Toc151366577)

[2.1. Introduction 10](#_Toc151366578)

[2.2. Objectives and purpose 10](#_Toc151366579)

[2.3. Scientific justification and rationale of study population 11](#_Toc151366580)

[3. Study design 12](#_Toc151366581)

[3.1. Primary and secondary endpoint 13](#_Toc151366582)

[3.1.1. Primary endpoint 13](#_Toc151366583)

[3.1.2. Secondary endpoints 13](#_Toc151366584)

[3.2. Measures to minimize bias 14](#_Toc151366585)

[3.3. Study duration and duration of participant’s participation 14](#_Toc151366586)

[3.3.1. Schedule of events 14](#_Toc151366587)

[3.4. Early termination of the study 14](#_Toc151366588)

[4. Selection of the study participants 14](#_Toc151366589)

[4.1. Study setting 14](#_Toc151366590)

[4.2. Recruitment 14](#_Toc151366591)

[4.3. Inclusion criteria 15](#_Toc151366592)

[4.4. Exclusion criteria 15](#_Toc151366593)

[4.5. Criteria for discontinuation of study 15](#_Toc151366594)

[4.5.1. Discontinuation of individual participants 15](#_Toc151366595)

[**5.** **Treatment of participants** 15](#_Toc151366596)

[5.1. Identity of investigational products 15](#_Toc151366597)

[5.1.1. Experimental intervention (treatment / medical device) 15](#_Toc151366598)

[5.1.2. Comparator 17](#_Toc151366599)

[6. Description of data management 17](#_Toc151366600)

[6.1. Specification of source documents 17](#_Toc151366601)

[6.2. Data management system 17](#_Toc151366602)

[6.3. Data security, access, archiving and back up 17](#_Toc151366603)

[7. Statistics 18](#_Toc151366604)

[7.1. Hypothesis 18](#_Toc151366605)

[7.2. Sample size calculation 18](#_Toc151366606)

[7.3. Description of statistical methods 18](#_Toc151366607)

[7.3.1. Datasets to be analysed, analysis populations 19](#_Toc151366608)

[7.3.2. Primary Analysis 19](#_Toc151366609)

[7.3.2.1. Secondary Analyses 19](#_Toc151366610)

[7.3.2.2. Interim analyses 19](#_Toc151366611)

[7.3.2.3. Deviation(s) from the original statistical plan 19](#_Toc151366612)

[7.4. Handling of data 19](#_Toc151366613)

[8. Duties of the Investigator 19](#_Toc151366614)

[8.1. Investigator’s confirmation 20](#_Toc151366615)

[Local regulations / Declaration of Helsinki 20](#_Toc151366616)

[Notification of safety and protective measures (HRO Art. 20) 20](#_Toc151366617)

[Serious events (HRO Art. 21) 20](#_Toc151366618)

[8.2. Project management 20](#_Toc151366619)

[9. Ethical considerations 20](#_Toc151366620)

[9.1. Independent Ethics Committee (IEC) 20](#_Toc151366621)

[9.2. Evaluation of the risk-benefit ratio 20](#_Toc151366622)

[9.3. Participant information and consent 20](#_Toc151366623)

[9.4. Registration of clinical trial 20](#_Toc151366624)

[9.5. Participant confidentiality 20](#_Toc151366625)

[9.6. Participants requiring particular protection 20](#_Toc151366626)

[9.7. Damage coverage 21](#_Toc151366627)

[9.8. Participant compensation 21](#_Toc151366628)

[9.9. Other aspects 21](#_Toc151366629)

[10. Quality control and quality assurance: description of measures 21](#_Toc151366630)

[10.1. Risk management 21](#_Toc151366631)

[10.1.1. Risk identification, assessment and mitigation 21](#_Toc151366632)

[10.1.2. Study-specific preventive measures 21](#_Toc151366633)

[10.2. Monitoring 21](#_Toc151366634)

[10.3. Audits and inspections 21](#_Toc151366635)

[10.4. Confidentiality, data protection 21](#_Toc151366636)

[10.5. Translations - Reference language 22](#_Toc151366637)

[10.6. Storage of biological material and related health data 22](#_Toc151366638)

[11. Dissemination of results and publication policy 22](#_Toc151366639)

[11.1. Dissemination to scientific community; incl. lead in publications 22](#_Toc151366640)

[11.2. Information of community and policy makers 22](#_Toc151366641)

[12. References 23](#_Toc151366642)

1. **Abbreviations**

| CHW | Community Health Worker |
| --- | --- |
| ECD | Early childhood development |
| ICF | Informed consent form |
| IEC | Independent ethics committee |
| IPA | Innovations for Poverty Action |
| IRB | Institutional review board |
| KFPE principles | Principles developed by the Commission for Research Partnerships with Developing Countries (KFPE) serve as Guide for Transboundary Research Partnerships |
| LMIC | Low- and middle income country |
| PC | Primary Care |
| SD | Standard deviation |
| Swiss TPH | Swiss Tropical and Public Health Institute |
| UTH | University Teaching Hospital in Lusaka |
| UNZA | University of Zambia |
| WCW | Wellbeing Community Workers |
| WHO | World Health Organization |
| HSCL-25 | Hopkins Symptoms Checklist-25 items |
| WHODAS2.0 | World Health Organization Disability Assessment |
| SRQ-20 | Self-Report Questionnaire |
| ACOK | Screen for Co-Occuring Disorders |
| PSYCHLOPS | Psychological Outcome Profiles |

1. **Synopsis**

| **Sponsor/Sponsor-Investigator** | Dr. Günther Fink |
| --- | --- |
| **Co-Principal Investigator** | Dr. Irene Falgas-Bague |
| **Study Title** | The impact of PM+FM intervention on improving mother’s mental health in Zambia |
| **Short Title/Study ID** | PM+FM |
| **Protocol Version and Date** | Version 1.1; 29.10.2022 |
| **Trial Registration** | Pending – will be registered on Clinicaltrials.gov |
| **Study Category with** **Rationale** | Other Clinical Trials, Category A  Rationale:  - Research project in which persons are prospectively assigned to a health-related intervention in order to investigate its effects on health or on the structure and function of the human body.  - Definition of Health (WHO): Health is a state of complete physical, mental and social well-being and not merely the absence of disease or infirmity |
| **Clinical Phase** | Not applicable. Not a clinical trial with drugs. |
| **Background and Rationale** | CMHD are particularly prevalent among women, posing a major threat to their own and their children’s wellbeing. In Zambia, our research team found that around 28% of women suffer from anxiety or depression.  Despite the high prevalence of CMHD and the existence of effective treatments, interventions supporting women living in low-resourced settings remain limited. Recent community-based programs have succeeded in cost-effectively tackling minor mental health problems, but have not helped individuals with severe symptoms.  This study builds on a parent study (Zamcharts), which identified a high prevalence of women with anxiety and/or depression. We propose to design, test and validate a community-based intervention nested within the public primary health system in Zambia, as a mental health care delivery platform to identify and treat women with mild-to-severe CMHDs. We aim to demonstrate the impact of integrating high-quality mental health care in the national system. |
| **Objective(s)** | To 1) adapt the Problem Management plus program to sub-Saharan mothers living in Zambia and to 2) assess the acceptability, feasibility and potential efficacy of the resulting intervention in reducing mental health symptoms and promoting positive maternal-child relationship among women taking care of young children in Lusaka, Zambia. |
| **Outcome (s)** | The primary study outcomes will be 1) number of sessions of PM+ attended (acceptance of the program = >50% of sessions attended; feasability within the local context = ≥ 1 session attended and offered and participants’ and providers’ qualitative feedback) and 2) improve their mental health status (overall mental health symptoms by SRQ-20 <7; Hopkins Symtpoms checklist reduction (% of cutoff <1.0.6),  Secondary outcomes: Patient Health Questionnaire (PHQ-9) (0-27; cutoff score, >10) for depressive symptoms; Generalized Anxiety Disorder Scale (GAD-7) score range, 0-21; cutoff score, >10) for generalized anxiety symptoms; Psychlops, psychological outcome profiles (designed to evaluate problem management plus intervention) and World bank toolkit on child-mother relationship. |
| **Study Design** | Randomised control trial |
| **Inclusion/Exclusion Criteria** | All mothers enrolled in Zamcharts clinical trial (n=790) with SRQ-20>7.  Exclusion criteria:   - Mothers who plan to migrate out of the study area within 6 months of the enrolment date - Mothers with severe substance use symptoms, bipolar disorder or squizophrenia - Mothers receiving psychotherapy elsewhere |
| **Measurements and Procedures** | From the total of 265 mothers residing in Lusaka district participating in the ZamCharts study and with mental health needs, we will divide them into two groups:  a control group a intervention group  All women will be called for a screening interview and then, if eligible, invited to receive a baseline interview followed by the intervention (provided in health facilities, by phone and at home) at the beginning and an endline interview at the end of the study.  Families in the intervention group will receive a locally adapted version of the Problem-Management Puls intervention developed by WHO after the baseline interview that allows them to better manage life stressors. The intervention will be provided by trained community health workers, started in groups (1^st^ session) and then continued individually by phone, or in-person at health facilities or at women’s home.  Severe cases will be referred to psychiatry services to evaluate need of antidepressants. |
| **Study Product/Intervention according to KlinV, if applicable** | This study will test an intervention: Problem Management+For Moms (PM+FM) program to reduce mental health symptoms among women and increase mother-child interaction |
| **Comparator(s) (if applicable)** | The comparator for the interventions is standard local caregiving (no intervention) among mothers participating in the Zamcharts clinical trial |
| **Study Duration** | 9 Months |
| **Participants** | A total of 265 caregivers and their children are expected to participate in the study. |
| **Study Schedule** | Sept. 2022: study setup, software development and IRB approval. Identification of WCW  Sept. 2022 – Nov. 2022: Training of 3-5 WCW  Nov. 2022: Enrollment of participants. Screening.  Dec. 2022 – April 2023: implementation of interventions  April 2023: Endline assessments  May 2023 - December 2023: Data analysis & dissemination |
| **Study Centre(s)** | The study will be coordinated by the PIs, and implemented by Innovations for Poverty Action Zambia in direct collaboration with the Zambian Ministry of Health and the department of psychiatry of the University Teaching Hospital in Lusaka. |
| **Statistical Considerations & Power calculations** | The study is statistically powered to detect a 0.25 SD improvement in SRQ-20 over an 6-month period between mothers receiving the PM+FM and mothers not receiving the intervention, assuming α=0.05, a follow-up rate of 85% (attrition < 15%), and an intra-class correlation coefficient (ICC) of 0.05 . The estimated improvement is based on the results from similar studies. |
| **Ethical Statement** | This study will be conducted in compliance with the protocol, the current version of the Declaration of Helsinki, KFPE principles for transboundary research as well as all national legal and regulatory requirements. |

# Background information

## Introduction

Mental health disorders affect millions of mothers in low-and-middle income countries (LMICs), limiting their ability to care for themselves and their children. Effective treatments for mental health disorders are widely available in high-income countries, but rarely applied in LMIC settings. Through this pilot, we aim to show that high-quality mental health support is possible in low-resource settings by combining community-based programming with targeted triaging to higher-level care. If successful, this evidence can provide a first step to improved mental health care in Zambia and many other settings.

Psychosocial programs integrated in PC systems increase access while providing treatment of minor CMHD within the community. However, in LIMC, the lack of supporting health policies and long-term resources limit their long-term effect. Moreover, they are not sufficient to treat severe cases. For those, pharmacological treatment is the most effective and considered the first treatment option but, in Zambia, systems are not in place to properly identify them and there is no data on the delivery of psychiatric medication within PC.

We propose to develop and pilot a mental health program and delivery platform for a set of interventions in Zambia to reduce the impact of CMHD disorders among women with young children, building upon the existing PC system and available evidence-based practices.

## Objectives and purpose

The long-term goal of this pilot study is to implement, if found effective, the EM-PM+ intervention within the primary care health system at the national level.

Primary objective**.** We aim to design, test and implement an evidence-based pilot mental health care program integrated within the primary health system for reducing mental health burden among mothers in Lusaka

Secondary objectives**.**

a. To evaluate the acceptability, feasibility and potential efficacy of a culturally sensible evidence-based intervention, the Expanded for Moms-Problem Management Plus (EM-PM+) in reducing mental health symptoms and promoting positive maternal-child relationship among women taking care of young children in Lusaka, Zambia compared to the best locally available care in reducing mental health burden among mothers in Lusaka and improving child development.

c. To build local capacity on mental health access in Lusaka for long-term impact.

e. To produce and disseminate high-quality international scientific publications with the highest ethical standards on the project findings to improve the evidence of mental health interventions in LMICs.

We hypothesize that 1) the EM-PM+ will be accepted by offered individuals (measured as >50% of sessions attended, satisfaction measure and qualitative feedback), 2) the EM-PM+ is feasible within the local context (measured by ≥ 1 session attended and offered and participants’ and providers’ qualitative feedback), 3) individuals receiving the EM-PM+ intervention will improve their wellbeing through improvement of mental health status and maternal-child relationship.

## Scientific justification and rationale of study population

As stated above, mental health disorders are highly prevalent worldwide and a leading cause of disability^1^. Depression and anxiety are the most common disorders with over 300 million cases worldwide. ^2,3^ Mental health disorders result from a complex interaction of social, psychological and biological factors and their development is related to chronic stress exposure as well as to acute trauma experiences. In Zambia, recent data from the our team suggest high rates of distress among women in charge of young children, however, common mental health disorders remain unnoticed and mainly untreated.^4^ The public healthcare system in Zambia, similar to other neighboring LMICs, offers only very limited mental health services. Existing mental health care and mental health workforce mainly focus on attending people with severe mental illness such as schizophrenia, other forms of psychosis and severe behavioral disorders. This care is concentrated in a few psychiatric institutions which are permanently full^5^. Undetected and untreated mental health problems n adversely affect women and their children in many ways, including reduced wellbeing and quality of life, increased food insecurity and reduced engagement with children, which can result in slower child development and an increased risk of anxiety, reduced physical growth, cognitive impairment^6^ and higher risks of non-adherence to HIV treatment for both.^7^ Zambia, faces several challenges in addressing such common mental health disorders within the primary care system. Shortage of mental health professionals, unmet training needs and lack of evidence-based psychosocial interventions adapted to the local context are currently the most important obstacles to serve this population. Evidence suggests that culturally adapted psychosocial programs, delivered by trained paraprofessionals or community health workers (CHWs), can be effectively integrated in healthcare systems, increasing mental health access and workforce capacity ^8,9^ while allowing the provision of evidence-based practice to treat common mental health disorders within the primary care setting along with other non-communicable diseases.^10^

In Zambia, preliminary data collected by our research team suggest that 28% of mothers are in mental distress, one quarter of whom have severe symptoms. Like in many other LMICs, mental health resources are limited and mainly concentrated in a few psychiatric institutions focused on caring for severe illnesses such as schizophrenia. Most CMHDs remain undiagnosed and untreated as primary care (PC) systems are not equipped to address mental health needs of the population.

Evidence suggests that culturally adapted psychosocial programs, delivered by community health workers (CHWs), can be integrated in healthcare systems, increasing mental health access and workforce capacitywhile allowing the provision of evidence-based practice to treat mild to moderate common mental health disorders within the primary care setting along with other non-communicable diseases. However, two important obstacles hinder the implementation of psychosocial interventions within most sub-Saharan regions, including Zambia: 1) the lack of available culturally adapted evidence-based interventions tackling women with small children, 2) the treatment of those with severe symptoms.

First, even though the proven effectiveness of some psychosocial interventions in treating mild to moderate mental health symptoms, there are no tested treatments adapted to women in charge of young children living in low income settings. Second, around 30% of people with CMHD suffer severe symptoms which heavily impair the capacity to develop daily tasks including low performance in parenting activities, domestic duties and self-care routines including their capacity to engage in psychosocial intervention sessions. For those cases, regardless of the economic development of the country, pharmacological treatment is the first treatment option. However, even though there are generic medications, at a low cost and available in all areas of the world there is very little data on the effectiveness, training in use and management of psychotropic medication within the primary health system of low-income settings.

Our proposed project, lead by a clinical psychiatrist collaborating with a local team of clinicians and policy makers, will overcome these challenges by 1) testing a multi-level intervention that adequately identifies and treats women with mental health symptoms, no matter their severity

The feasibility, acceptability and effectiveness of this multi-level intervention will be pilot tested through a clinical trial design.

# Study design

We propose to conduct a trial with mothers in Lusaka district. This area was selected to ensure feasibility of the study. Lusaka is an urban area with relatively easy access to care and transportation.

This is a pilot study nested within the “THE IMPACT OF GROWTHCHARTS ON STUNTING AND CAREGIVER AWARENESS: RESEARCH AND POLICY ENGAGEMENT FOR POTENTIAL SCALING (REF. NO. 1411-2020)” clinical trial using a mix-methods analysis. 790 caregiver-child dyads were enrolled in this study in Lusaka in 2021; 30% of these women displayed mild to severe symptoms of depression and/or anxiety (SRQ-20 ≥ 8). For this feasibility study, we will randomly select women from this subgroup. All selected women will be contacted and reassessed with the Self-Reporting Questionnaire (SRQ-20) and further assessed using the Hopkins Symptoms Checklist-25 items^11-13^.

Final ***eligibility*** will be determined by 1) SRQ-20 >7 (clinically significant symptoms of depression or/anxiety anxiety), 2) women with 1 hour a week availability not planning to move within the next 6 months, 3) capacity to consent and 4) not receiving current mental health care. Individuals with active suicidal ideation (determined by Paykel scale^14^), severe substance use (ACOK-SUD >4),^15^ mania or psychotic symptoms^16^ will be excluded and referred to primary care provider or partnering psychiatrist (for emergency cases). We expect that around 70 caregivers will meet the inclusion criteria for the mental health feasibility study. All selected women from the intervention group will be contacted and reassessed with the Self-Reporting Questionnaire (SRQ-20) and the Hopkins Symptoms Checklist 25.

Eligible individuals will continue with a Baseline interview to assess main outcome measures. We plan to focus on engaging participants in each of the research components of the trial (assessments and sessions) through systematic engagement protocol including regular calls to participants and home visits after two weeks of non-responsiveness. All eligible subjects will be randomized with probability of 0.5 to either treatment or control group. Randomization will be based on a random draw generated by the senior author (GF) using the Stata SE 16.0 statistical software package.

Each enrolled participant will be assigned a specifically trained CHW, that we will call Wellbeing-Community Worker (WCW). WCWs will conduct a welcome call to their assigned participant and provide 10-12 individual sessions of the PM+FM intervention.

In a first step, we will assess efficacy by re-evaluating mental health symptoms (using HSCL-25) of caregivers again 5 months after the intervention. In a second step, we will compare changes in mental health at the population level using data from the larger study. Specifically, we will use endline data to be collected in the second quarter of 2023 to look at mid-term mental health outcomes (SRQ-20) as well as caregiver-child interactions and child developmental outcomes.

Qualitative data will be collected through semi-structured interview to participants during treatment completion assessment (5 months after enrollment) and focus groups with WCWs providing the intervention (analyzed using thematic approach to inform and complement quantitative results on feasibility, acceptability, and efficacy of the PM+FM intervention.

## Primary and secondary endpoint

### Primary endpoint

The primary endpoint will be SRQ-20 = or < 7 6 months after enrollment in the trial and/or HSCL-25 <1.06 6 months after enrollment.

We will also collect data on attendance rates to assess feasibility and acceptability throughout the deployment of the intervention:

- Feasibility will be assessed by the percentage of participants completing 1 or more sessions of the intervention and through a focus group with the intervention providers.
- Acceptability will be evaluated using the percentage of participants completing the program (≥50% sessions completed) together with qualitative feedback from participants and providers through a semi-structured interview at the end of the intervention and participants’ feedback from a semi-structured interview at the end of the intervention.

### Secondary endpoints

Secondary endpoints will be the psychological outcome profiles to measure women’s wellbeing and mother-child interaction and child early development.

| **Table 1. Measures and Outcomes** | **Measures Exploratory Efficacy Outcome** |
| --- | --- |
| **Screener measures** | Mania and Psychosis 4 question history (+ MDQ if positive), Capacity to consent (using a validated screening tool), ACOK (> 1 scores for MH and <3 SUD), Paykel suicidality risk scale -.  SRQ-20 >7 |
| **Primary Outcome Measures for EM-PM+ (assessed at enrollment, and 6 months** | |
| **SRQ-20** | 20 items measuring risk of common mental health problem. Used in the intent-to-treat analysis to explore effectiveness of the intervention using control group (endline data from parent study) |
| **Feasibility** | % of 1 or more sessions completed. Completion rates; ease/burden. Qualitative feedback from participants and providers. |
| **Acceptability** | 1) Participation rates; 2) Satisfaction scale; 3) Relevance of PM+FM. Qualitative feedback from participants and providers. |
| **Secondary Outcomes** | |
| **PSYCHLOPS** | Psychological Outcome Profiles (5 items) |
| **WHO-DAS2** | Functionality |
| **HSCL-25** | 10 questions anxiety, 15 depression; (α = 0.90). Used for the pre-post treatment analysis. |
| **World Bank’s** Toolkit **and** Inventory^17^ | Selected measures from the World Bank Toolkit to address mother-child interactions and child early development. |

## Measures to minimize bias

This study will be conducted as a randomized controlled trial, which should minimize the risk of selection and confounding bias. Women’s wellbeing will be assessed objectively through trained interviewers blinded by the treatment condition minimizing the risk of reporting or social desirability biases.

## Study duration and duration of participant’s participation

The project will be divided into three phases: A preparatory phase (Month 1 - 6) will primarily focus on developing and adjusting the intervention, and for piloting field logistics. This initial phase will be followed by a trial phase (Month 7-12) and a final analysis and dissemination phase (year 2).

We anticipate to start identification cases, screening and enrollment to the trial in October- Nov. 2022. An Endline assessment will be conducted approximately 5 months after completion of the baseline survey (April 2022).

### Schedule of events

Table 1. Project Milestone

| Date | Description |
| --- | --- |
| July 2022 – Sept. 2022 | Study setup, IRB approval, identification of WHW, |
| Sept. 2022 – Oct. 2022 | Training of 4-6 WCW. Identification of participants (Screening) |
| Nov. 2022 – Mar 2023 | Baseline and Intervention period |
| May 2023 - June 2023 | Endline assessments |
| Jul 2023 – Sept. 2023 | Data analysis |
| Oct. 2023 – Dec. 2023 | Study dissemination |

## Early termination of the study

We do not foresee any events that would result in an early termination of the study.

# Selection of the study participants

## Study setting

This study will be conducted in Lusaka district of Zambia

## Recruitment

All women participating in Zambcharts parent study complying with the inclusion criteria and living in the enumeration areas (each enumeration area contains 200-500 households, and correspond to villages in rural settings and blocks in urban areas) of Lusaka district will be invited to participate in our study. Participants will be contacted by trained study staff, who will explain the study to caregivers and enroll them into the study conditional on their consent.

We plan to focus on engaging participants in each of the research components of the trial (assessments and sessions) through systematic engagement protocol including regular calls to participants and home visits after two weeks of non-responsiveness.

## Inclusion criteria

1) SRQ-20 >7 (clinically significant symptoms of depression or/anxiety anxiety), 2) women with 1 hour a week availability not planning to move within the next 6 months, 3) capacity to consent and 4) not receiving current mental health care.

## Exclusion criteria

Women with active suicidal ideation (determined by Paykel scale^14^), severe substance use (ACOK-SUD >4),^15^ mania or psychotic symptoms^16^ will be excluded and referred to primary care provider or partnering psychiatrist (for emergency cases). Additionally, women planning to move in the next 6 months were excluded from the study.

We expect that around 70 caregivers will meet the inclusion criteria for the mental health feasibility study.

All eligible subjects will be randomized with probability of 0.5 to either treatment or control group. Randomization will be based on a random draw generated by the senior author (GF) using the Stata SE 16.0 statistical software package.

## Criteria for discontinuation of study

We do not foresee any circumstances under which the trial would be discontinued.

### Discontinuation of individual participants

It is possible that some caregivers will not want to (consent to) participate in the endline survey. Based on previous studies in this area, we believe this risk to be small. We have allowed for 15% attrition (mostly due to migration) between the baseline and endline survey.

1. **Treatment of participants**

## Identity of investigational products

### Experimental intervention (treatment / medical device)

To develop the PM+FM intervention, we followed the Castro-Barrera framework for cultural adaptation of evidence-based interventions. ^23^ Therefore, the intervention is culturally adapted to women’s local context of social and cultural values and norms.

This study aims at assessing one intervention: Problem Management Program-For Moms

**Intervention:** The PM+FM intervention is a cognitive-behavioral therapy-based transdiagnostic psychosocial intervention using a context-adapted version of the PM+ manual from WHO and specific sections from the Strong Minds psychosocial intervention and psychoeducation material from a psychosocial stimulation intervention in early childhood to improve women’s wellbeing including mental health symptoms and mother-child relationship. The intervention:

- Is provided individually, organized in 10 to 12 1-hour sessions and provided on a weekly basis by a specifically trained and supervised WCW in women’s homes.
- Lasts a maximum of 5 months. Following regular clinical patterns of attendance, participants can schedule the sessions within this period to finish the program.
- Will be culturally adapted to women’s local context of social and cultural values and norms using a systematic cultural adaptation framework. To develop the PM+FM intervention, we followed the Castro-Barrera framework for cultural adaptation of evidence-based interventions.^18^ Therefore, the intervention is culturally adapted to women’s local context of social and cultural values and norms.
- Follows a collaborative and patient-centered approach where individual’s needs are central and contents of the intervention are adapted to each person.
- Combines psychoeducation, problem-solving and cognitive restructuring techniques, motivational interviewing approach, effective communication practice and mindfulness practice.

| **PM+FM Intervention summary of contents** | |
| --- | --- |
| Session 1 | Symptom’s assessment. Introduction of the program and confidentiality. Cultural Formulation interview to understand woman’s need. Psychoeducation about mental health needs (understanding adversity) and maternal-child positive relationship. Relaxing breathing. |
| Session 2 | Barriers to change, managing problems and stress management strategies. Introduction to mindfulness. |
| Session 3 | Symptom’s assessment. Managing Problems (Problem-solving technique), “get going, keep doing” strategies (Behavioral activation). Managing Stress strategy and mindfulness practice. |
| Session 4 | Managing Problems: understanding unhelpful thoughts. Positive upbringing strategies. Stress management and mindfulness practice. |
| Session 5 | Symptom’s assessment. Importance of support for positive upbringing and stress management. Strengthening social support. Mindfulness practice. |
| Session 6 | Effective communication skills, Mindfulness practice |
| Session 7 | Symptoms’ assessment. Improving healthy habits for me and my child (nutrition, sleep, and substance use). Mindfulness practice. |
| Session 8 | Positive thinking and self-compassion practice. Mindfulness practice. |
| Session 9 | Review of the techniques. Staying Well and Looking to the future (Self-care plan). Mindfulness practice. |
| Session 10 | Review of self-care plan. Social resources and referral. Mindfulness practice. Closure and Ending the programme. |

- 2 booster sessions can be delivered to accommodate urgent needs and/or consolidate core content of the program (i.e. problem management and self-care plan).
- **Management of Severe cases:** Cases found to be severe in mental health symptoms or substance use symptoms, will be referred to our collaborating team of psychiatrists and psychologists from UTH in Lusaka, lead by Dr. Paul. Medication prescription and monitoring will be tracked by the research team. Pharmacological treatment (indicated by the psychiatrist) will be subsidized by the project in case participant cannot afford it.
- **Training and Supervision of WHW:** We will identify 4 to 6 people with at least a high school diploma, with some previous experience of working within the community and interested in mental health topics to be trained as WCWs. The training program occur in 4 full days of instruction followed by 4 weeks of role-plays with 2 hours weekly group supervision. Instructional training starts by addressing program objectives, ethics, emergency protocol procedures, psychoeducation about common mental health problems (day 1) and basic components of psychotherapy (i.e. motivational interviewing, cultural formulation, mindfulness practice and problem solving technique (Day 2). During the third and fourth day of instructional training, we will focus on the specific sessions’ content, special clinical situations (i.e. substance use involvement, domestic violence, and suicidal thoughts), and training related to their integration in the clinical site, including communication and referral procedures. It will end with a review of the emergency protocol procedures. Instructional trainings will be videotaped for further use for needed subsequent trainings. After roleplaying with two example cases and receiving weekly supervision. WCWs will deliver the intervention to two first cases as pilots. Pilot cases (ineligible for the trial because of mild symptoms or because already receiving treatment). All role-plays and pilot sessions will be audiotaped, transcribed and heard by the supervisor. The **supervision protocol** includes weekly individual and group supervision throughout the training process (individual supervision) and trial (group supervision) to support the WCWs and provide feedback on the session tapes, share the fidelity forms and to resolve issues related to the delivery (e.g. severe cases and drifting). We plan to collect data on training needs and mental health basic knowledge among the WCWs at the moment of hiring and every 6 months to evaluate mental health care delivery capacity. The supervisor will be a local psychologist specially trained by the PI of this project.

### Comparator

The comparator (control group) will receive no intervention.

# Description of data management

## Specification of source documents

All source documents will be created in English. Data collection tools (screening, baseline and endline survey forms and informed consent forms) will be translated to local language. Back-translations will be made to ensure accuracy of translations.

## Data management system

All data will be collected and stored electronically using the Survey Collect (SurveyCTO) software. Survey CTO is tool intended to facilitate mobile data collection services. It consists of an Android app that replaces paper forms used in survey-based data gathering. It supports a wide range of questions and answer types and can be utilized without network connectivity. All data collection will occur offline on password protected Android devices. Data connectivity will only be used to send data collected in the field. Data storage will be on a local secured computer in Zambia and on ServeyCTO’s secure servers.

Data storage will be on a local secured server at IPA Zambia.

## Data security, access, archiving and back up

Project data is only accessible to authorised personnel who require data to fulfil their duties within the scope of the research project. On the eCFRs and other project specific documents, participants are only identified by a unique participant number. Data and databases will not be shared with the public and will only be available (fully anonymised) for verification purposes of authorities or scientific journals (as condition to publish results) – on request only. All study data will be archived for a minimum of ten years after study completion.

# Statistics

## Hypothesis

In this sub-project of the ZamCharts trial, we wish to assess the following three hypotheses

1) the PM+FM will be accepted by offered individuals (measured as >50% of sessions attended, satisfaction measure and qualitative feedback),

2) the PM+FM is feasible within the local context (measured by ≥ 1 session attended and offered and participants’ and providers’ qualitative feedback),

3) individuals receiving the PM+FM intervention will improve their wellbeing through improvement of mental health status and maternal-child relationship.

## 7.2. Sample size calculation

This is a pilot study nested within the “The impact of growthcharts on stunting and caregiver awareness: research and policy engagement for potential scaling (ref. no. 1411-2020)” clinical trial. 800 caregiver-child dyads were enrolled in this study in Lusaka in 2021; 28% of these women displayed mild to severe symptoms of depression and/or anxiety (SRQ-20 ≥ 8). For this feasibility study, 30% of the women enrolled in the parent trial displayed mild to severe symptoms of depression and/or anxiety (SRQ-20 ≥8). For this study, we expect an attrition rate of 13% based on previous studies. With an expected analytical sample size of 100 women per arm we are powered to detect an intent-to-treat difference of 20 percentage points in the prevalence of depression at endline with power 0.8, and 23 percentage point difference in depression with power 0.9. We are aware that for the current trial we are not powered to detect small effect size. Thus, we will only explore the efficacy of the intervention to generate some preliminary efficacy estimates that will serve us to power and elucidate the needed sample size for a larger effectiveness trial.

## Description of statistical methods

First, we will begin with descriptive analysis of all data by site, using means, variances, quartiles, and graphical assessments of the distribution and skewness of the data. We will describe feasibility and acceptability measures at the participant level.

Then, we will proceed with a pre-post intervention analysis using the Hopkins Symptoms Checklist as a main outcome to compare mental health symptoms before and after receiving the intervention. Moreover, on a second phase, we will calculate effect sizes and trends of effectiveness between intervention group and the non-treated group from the parent trial using endline data from parent trial.

We will use standard linear regression models to assess the impact of the intervention arm on mother’s mental health and mother-child relationship. The intervention outcome variable will be SRQ-20 used as a dichotomous variable with the cutoff at seven. Separate models will be estimated with and without baseline covariates.

The feasibility, acceptability, and difficulties and successes in carrying out research and intervention activities will be explored through a mix-methods analysis including dosage analysis (attendance to the intervention’s sessions) and semi-structured interviews with the participants and focus group with intervention’s providers. The burden of completing the assessments and PM+FM on the time and effort of participants, satisfaction with the intervention, and barriers and facilitators to adherence will be explored through semi-structured interviews with all the participants enrolled in the intervention arm (including participants that have dropped out). Qualitative data resulting from the open-ended questions to participants and focus groups with WCHWs will be transcribed, coded and analysed using a thematic approach to inform on feasibility, acceptability, and explain the potential efficacy of the PM+FM intervention

### Datasets to be analysed, analysis populations

The final analysis will primarily rely on the data collected in the endline survey. Baseline variables will be used to 1) assess the extent to which balance was reached through the randomization and 2) create a set of control variables used in adjusted models.

### Primary Analysis

The primary analysis of program feasibility, acceptability and pre-post treatment analysis will be conducted directly be the PI of the parent study (GF) and the present sub-study (IFB) with support from the co-investigators. Effectiveness analysis will be done by the PI of the parent study (CF). This analysis will be intention-to-treat, and thus not adjust for protocol compliance.

#### Secondary Analyses

Secondary analysis focusing on subgroup effects as well as looking at secondary outcomes (mother-child relationship, functionality) will be conducted by the research team. We will also conduct per-protocol analysis to assess program impact among compliant caregivers.

#### Interim analyses

No interim analysis is foreseen.

#### Deviation(s) from the original statistical plan

Any deviations from the original statistical plan will be reported in the Methods section of the final project report.

## Handling of data

Missing data will arise when a participant refuses to answer a question or complete the part of the examinations. Missing data may also arise is a participant drops out of the study. All participants are informed that their participation is completely voluntary and that they may refuse to answer any of the questions asked and can stop the evaluation at any time as well as discontinue participation at any time without any consequences. Missing data on covariates will be imputed using multiple imputations using chained equations. Missing outcome data due to sample attrition will not be imputed; however, we will conduct and report analyses examining the extent to which attrition differed across treatment conditions.

# Duties of the Investigator

## Investigator’s confirmation

### Local regulations / Declaration of Helsinki

This research project will be conducted in accordance with the protocol, the Declaration of Helsinki, KFPE principles for transboundary research as well as all national legal and regulatory requirements.

### Notification of safety and protective measures (HRO Art. 20)

see 10.2.

### Serious events (HRO Art. 21)

see 10.2.

## Project management

The PIs of the project will be responsible for management and coordination of the project.

# Ethical considerations

## Independent Ethics Committee (IEC)

All study documents will be reviewed by the EKNZ in Switzerland as well as the ethics board at the University of Zambia in Lusaka.

## Evaluation of the risk-benefit ratio

Given that the interventions are primarily designed to support mothers, adverse outcomes seem unlikely.

## Participant information and consent

Trained interviewers will explain the nature of the study to the participant, and answer all questions regarding this study, prior to obtaining informed consent.

Withdrawal and discontinuation

Participation is completely voluntary and participants may withdraw from the study at any time or may refuse to answer any questions without having repercussions on their participation. If the participant decides to withdraw from the study they may request that their information already obtained will be destroyed and electronic data deleted and not be used for evaluation.

## Registration of clinical trial

This trial will be registered at www.clinicaltrials.gov. We will also submit this study protocol for publication.

## Participant confidentiality

All records will be collected electronically and stored on a secure server. For analysis, all personal information will be removed, and a unique Participant Number (e.g. 2738) assigned to each participant. The Investigators will keep a separate confidential enrolment log that matches identifying codes with the participants’ names and residencies, preferably in the Trial Master File.

## Participants requiring particular protection

NA

## Damage coverage

NA

## Participant compensation

All participants will be given a small present (a piece of soap or something similar) to thank them for their time spent with interviews at the end of the interview. No incentives will be provided to attend to the intervention sessions.

## Other aspects

NA.

# Quality control and quality assurance: description of measures

All study staff will be carefully trained by the research team to ensure highest possible data quality. Data will be collected electronically, and reviewed for consistency on a weekly basis.

All field staff undergoes training in ethics in research.

## Risk management

### Risk identification, assessment and mitigation

The proposed interventions do not pose any known risk to participating women. Similarly, if women are not interested in attending the intervention, they can simply inform study staff they are no longer interested in receiving them.

### Study-specific preventive measures

NA.

## Monitoring

Both baseline and endline surveys will be monitored electronically through the Survey CTO software package.

## Audits and inspections

The study documentation and the source data/documents are accessible to the ethics committees and auditors/inspectors at all times. All involved parties will keep the participant data strictly confidential.

## Confidentiality, data protection

Project data is only accessible to authorized personnel who require data to fulfil their duties within the scope of the research project. On the eCFRs and other project specific documents, participants are only identified by a unique participant number. De-identified data will be made publicly available after the publication of the trial.

## Translations - Reference language

The reference language for all study documents is English. ICFs and CRFs (baseline and endline survey questionnaires) will be translated to local language.

## Storage of biological material and related health data

No biological material will be collected as part of this project. All data collected will be coded. Codes can be linked to personal identifying information including first and family name, address, phone number, e-mail address, village leader name. This will be stored using the Survey CTO server and only senior research team members (project manager, data manager and PI’s) affiliated to the study will have access to this database. Our project does not include the storage of bio-specimens.

# Dissemination of results and publication policy

## Dissemination to scientific community; incl. lead in publications

Data analysis will lead to a series of synthesizing reports and publications in peer-review scientific journals for which all principles of data safety and protection are considered, and any results are presented in a fully anonymized manner.

## Information of community and policy makers

This study will be conducted in close collaboration with the **Zambian Ministry of Health (MoH).** Dr. Mwape will be part of the advisory board along with local team of clinicians and policy makers.

Progress will also be shared through the MoH’s health sector mental health technically working group quarterly meetings.

# 12. References

1. Groce NE. Global disability: an emerging issue. *The Lancet Global Health.* 2018;6(7):e724-e725.

2. Baxter AJ, Scott KM, Vos T, Whiteford HA. Global prevalence of anxiety disorders: a systematic review and meta-regression. *Psychological medicine.* 2013;43(5):897-910.

3. Ormel J, VonKorff M, Ustun TB, Pini S, Korten A, Oldehinkel T. Common mental disorders and disability across cultures: results from the WHO Collaborative Study on Psychological Problems in General Health Care. *Jama.* 1994;272(22):1741-1748.

4. Mayeya J, Chazulwa R, Mayeya PN, et al. Zambia mental health country profile. *International Review of Psychiatry.* 2004;16(1-2):63-72.

5. Mwape L, Sikwese A, Kapungwe A, et al. Integrating mental health into primary health care in Zambia: a care provider's perspective. *International Journal of Mental Health Systems.* 2010;4(1):1-9.

6. Nyseth CA. The adverse effects of maternal depression and poverty on child development. 2015.

7. Wachs TD, Black MM, Engle PL. Maternal depression: a global threat to children’s health, development, and behavior and to human rights. *Child Development Perspectives.* 2009;3(1):51-59.

8. Balasubramanian BA, Cohen DJ, Jetelina KK, et al. Outcomes of integrated behavioral health with primary care. *The Journal of the American Board of Family Medicine.* 2017;30(2):130-139.

9. Vogel ME, Kanzler KE, Aikens JE, Goodie JL. Integration of behavioral health and primary care: Current knowledge and future directions. *Journal of Behavioral Medicine.* 2017;40(1):69-84.

10. Hoeft TJ, Fortney JC, Patel V, Unützer J. Task‐sharing approaches to improve mental health care in rural and other low‐resource settings: a systematic review. *The Journal of rural health.* 2018;34(1):48-62.

11. Ventevogel P, De Vries G, Scholte WF, et al. Properties of the Hopkins Symptom Checklist-25 (HSCL-25) and the Self-Reporting Questionnaire (SRQ-20) as screening instruments used in primary care in Afghanistan. *Social psychiatry and psychiatric epidemiology.* 2007;42(4):328-335.

12. Kaaya SF, Fawzi MS, Mbwambo J, Lee B, Msamanga GI, Fawzi W. Validity of the Hopkins Symptom Checklist‐25 amongst HIV‐positive pregnant women in Tanzania. *Acta Psychiatrica Scandinavica.* 2002;106(1):9-19.

13. Sandanger I, Moum T, Ingebrigtsen G, Dalgard OS, Sørensen T, Bruusgaard D. Concordance between symptom screening and diagnostic procedure: the Hopkins Symptom Checklist-25 and the Composite International Diagnostic Interview I. *Social psychiatry and psychiatric epidemiology.* 1998;33(7):345-354.

14. Paykel E, Myers J, Lindenthal J, Tanner J. Suicidal feelings in the general population: a prevalence study. *The British Journal of Psychiatry.* 1974;124(582):460-469.

15. Rubinsky AD, Dawson DA, Williams EC, Kivlahan DR, Bradley KA. AUDIT‐C scores as a scaled marker of mean daily drinking, alcohol use disorder severity, and probability of alcohol dependence in a US general population sample of drinkers. *Alcoholism: Clinical and Experimental Research.* 2013;37(8):1380-1390.

16. Unützer J, Katon W, Williams Jr JW, et al. Improving primary care for depression in late life: the design of a multicenter randomized trial. *Medical care.* 2001:785-799.

17. Fernald LC, Prado E, Kariger P, Raikes A. A toolkit for measuring early childhood development in low and middle-income countries. 2017.

18. Barrera Jr M, Castro FG, Strycker LA, Toobert DJ. Cultural adaptations of behavioral health interventions: A progress report. Journal of Consulting and Clinical Psychology. 2012;81(2):196-205.
